# Interplay between Structural, Electronic, and Topological Properties in Low-Dimensional Tellurium

**DOI:** 10.1021/acsomega.5c12108

**Published:** 2026-03-06

**Authors:** Gabriel Elyas Gama Araújo, Andreia Luisa da Rosa

**Affiliations:** Federal University of Goiás, Institute of Physics, Campus Samambaia, Goiânia 74960600, Brazil

## Abstract

We present a comprehensive first-principles investigation of the structural, electronic, vibrational, and topological properties of tellurium across its dimensional hierarchy, including bulk trigonal Te–I, two-dimensional tellurene polymorphs, and one-dimensional helical nanowires. Using density functional theory with full inclusion of spin–orbit coupling, we confirm that bulk Te–I is a narrow-gap semiconductor hosting Weyl nodes arising from broken inversion symmetry and degenerate phonon modes suggestive of chiral phonon behavior. In contrast, two-dimensional α- and β-tellurene are found to be topologically trivial ( $Z_{2}=0$ ), with no spin–orbit-driven band inversion in the occupied manifold. Beyond these established phases, we find that buckled kagome and buckled square tellurene lattices exhibit a nontrivial two-dimensional $Z_{2}=1$ topology of the occupied electronic bands, indicating incipient quantum spin Hall character in metallic systems. In contrast, one-sided hydrogen-passivated hexagonal tellurene realizes a fully gapped quantum spin Hall phase with a robust $Z_{2}=1$ invariant, preserved under applied strain and chemical functionalization. In the one-dimensional limit, helical tellurium nanowires preserve chirality and host edge-localized states accompanied by pronounced anisotropy in carrier effective masses. These results establish tellurium as a highly tunable platform for engineering topological phenomena across dimensionality, bridging three-dimensional Weyl physics, two-dimensional quantum spin Hall and incipient $Z_{2}$ phases, and one-dimensional helical systems.

## Graphical abstract

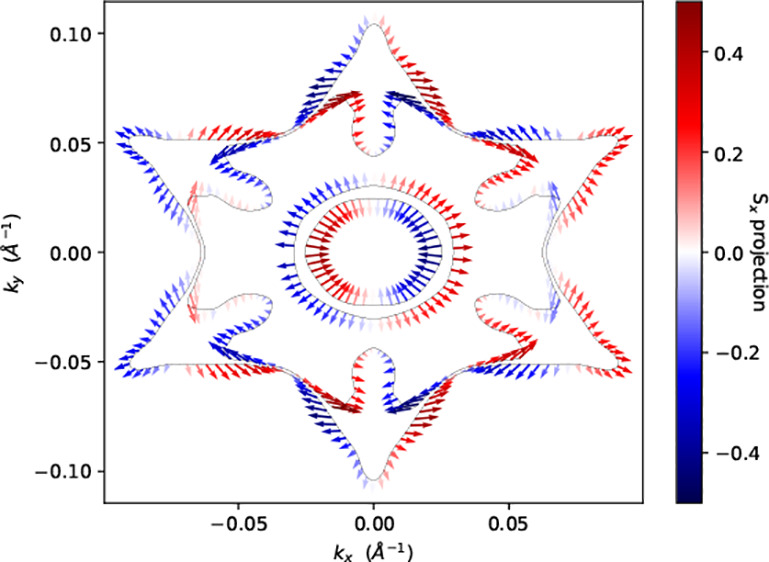

## 1 Introduction

Tellurium (Te) is a rare, silvery metalloid found only in trace quantities in Earth’s crust and seawater. Despite its scarcity, Te has attracted increasing attention owing to its distinctive physical and chemical properties, particularly as a semiconductor with emerging technological relevance. As a group-16 chalcogen, Te exhibits remarkable crystalline and electronic characteristics, including nontrivial topological behavior driven by strong spin–orbit coupling (SOC).^1,2^

In its bulk phase, Te crystallizes in a trigonal structure composed of one-dimensional (1D) chiral helical chains aligned along the *c*-axis. This noncentrosymmetric motif breaks inversion symmetry and gives rise to a rich landscape of topological phenomena. Trigonal tellurium is a narrow-gap semiconductor widely recognized as hosting Weyl nodes in its bulk band structure.^3–5^ The characteristic hedgehog spin texture in momentum space further emphasizes the critical role of inversion-symmetry breaking and SOC in shaping Te electronic topology.^2^

While bulk trigonal Te is now well understood, the evolution of topology in its low-dimensional derivatives remains less systematically understood across dimensionality. The two-dimensional α-phase ( $P\overset{-}{3}m1$ ) and monoclinic β-phase (*P*2/*m*) were first predicted theoretically^6^ and later synthesized on GaAs^7,8^ and graphene/6H-SiC(0001)^9^ substrates, respectively. Additional 2D allotropes have since been proposed and, in some cases, realized, including a rectangular phase on Ni(111)^10^ and a honeycomb tellurene lattice exhibiting an SOC-induced Dirac gap.^11^ These reduced-dimensional phases provide fertile ground for exploring tunable topological states through strain, substrate interaction, or chemical functionalization.

Tellurium can also form one-dimensional nanostructures such as nanowires and nanoribbons. First synthesized via hydrothermal methods,^12^ these systems preserve the helical atomic backbone of bulk Te, offering an ideal platform to probe chirality-driven physics under quantum confinement. The recent fabrication of ultrathin, high-crystallinity Te nanowires^13^ underscores the growing interest in identifying low-dimensional electronic and boundary-state phenomena in 1D Te architectures.

The interplay of structural flexibility, chirality, strong SOC, and symmetry breaking thus positions tellurium as a uniquely versatile platform for realizing and engineering emergent topological phases.^14,15^

Despite these advances, a unified understanding of how topology evolves across Te dimensionalities remains incomplete. Here, we address this gap through first-principles calculations of tellurium in its 3D, 2D, and 1D forms, elucidating how chirality, symmetry breaking, and SOC govern its electronic topology. We confirm the existence of Weyl nodes in bulk Te, reveal that 2D allotropes exhibit symmetry-tunable electronic structures, and demonstrate that helical Te nanowires retain bulk chirality while hosting edge-localized states associated with quantum confinement.

This work establishes symmetry-driven connections between the Weyl physics of bulk Te and its low-dimensional derivatives. Beyond advancing the fundamental understanding of symmetry-protected states in reduced dimensions, our findings highlight tellurium as a promising candidate for next-generation spintronic, optoelectronic, and quantum devices, where topological phases can be engineered via strain, confinement, and chemical modification.

## 2 Computational Details

Our investigation employs first-principles calculations performed with the Vienna Ab initio Simulation Package (VASP).^16,17^ The exchange–correlation potential is treated using both the generalized gradient approximation (GGA), parametrized by Perdew, Burke, and Ernzerhof (PBE),^18^ and the hybrid Heyd–Scuseria–Ernzerhof (HSE06) functional.^19^ Long-range van der Waals (vdW) interactions are included via the DFT-D3 correction method proposed by Grimme.^20^ Core–valence electron interactions are described using the projector augmented-wave (PAW) method,^21^ and the Kohn–Sham single-electron wave functions are expanded in a plane-wave basis set with a kinetic energy cutoff of 520 eV.

All atomic structures are fully relaxed until the residual forces on each atom are smaller than 1 × 10^–6^ eV/Å. To avoid spurious interactions arising from periodic boundary conditions, a vacuum spacing of 12 Å is introduced—along the *z*-direction for monolayers and along the transverse (*x* and *y*) directions for nanowires. The Brillouin zone is sampled using **k**-point meshes generated by the Monkhorst–Pack scheme,^22^ with grids of (15 × 15 × 8) for bulk Te, (13 × 17 × 1) for α-tellurene, (9 × 12 × 1) for β-tellurene, (13 × 17 × 1) for hexagonal planar, (24 × 24 × 1) for hexagonal buckled, (13 × 13 × 1) for pentagonal, (17 × 17 × 1) for Lieb-like, (21 × 21 × 1) for planar kagome, (19 × 19 × 1) for buckled kagome, (23 × 23 × 1) for square planar, (23 × 23 × 1) for buckled square, and (1 × 1 × 18) for nanowires.

Vibrational and thermodynamic properties are evaluated from phonon dispersion calculations using the finite-displacement method. Supercells of sizes (4 × 4 × 4) for bulk, (4 × 4 × 1) for monolayers, and (1 × 1 × 15) for nanowires are employed. The same plane-wave cutoff energy (520 eV) and **k**-point densities as in the electronic structure calculations are used, with a force convergence threshold of 10^–6^ eV/Å. Phonon dispersion relations and corresponding density of states (DOS) are computed using the Phonopy package.^23,24^

To evaluate the Chern number and the $Z_{2}$ topological invariant, maximally localized Wannier functions (MLWFs) are constructed using the Wannier90 package.^25^ The corresponding topological invariants are subsequently computed with the WannierTools postprocessing code.^26^ The topological properties of the one-dimensional nanowire were analyzed using the modern theory of polarization. Atomic structures are visualized using the VESTA software,^27^ and all plots and graphical analyses are produced with the Matplotlib library.^28^

## 3 Results and Discussion

Tellurium crystallizes in a stable trigonal phase composed of one-dimensional helical chains of Te atoms along the *c*-axis. Each atom has a 5*s* ^2^5*p* ^4^ configuration, where two 5*p* electrons form covalent bonds within the chains, while the 5*s* electrons remain core-like. The remaining 5*p* electrons form lone pairs oriented between chains, leading to interchain vdW interactions. This results in strong intrachain bonding and a quasi-layered, anisotropic structure.^29,30^

The helical structure exhibits structural chirality, adopting either the right-handed *P*3_1_21 (*D*_3_^4^) or the left-handed *P*3_2_21 (*D*_3_^6^) space group, making them noncentrosymmetric systems. After structure optimization shown in Figure 1, the obtained lattice parameters within GGA-PBE are *a* = *b* = 4.41 Å and *c* = 5.93 Å, with a Te–Te bond length of *d*_*h*_ = 2.90 Å. These values are very close to previous theoretical calculations^6–32^ and close to the experimental values of *a* = *b* = 4.45 Å and *c* = 5.93 Å.^33^

**Figure 1:**
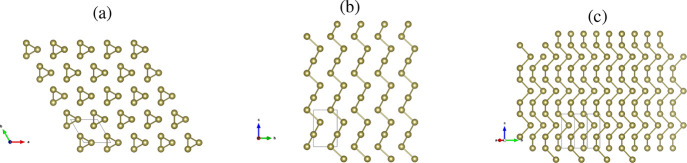
Relaxed geometry of trigonal Te–I: (a) top view projected along $\vec{c}$ ; (b) along $\vec{a}$ ; (c) perpendicular to $\vec{b}$ .

The dynamic stability of trigonal tellurium (Te–I) is investigated through phonon dispersion calculations within the harmonic approximation, employing the finite displacement method. The calculated phonon band structure of trigonal tellurium (Te–I) along with thermal properties are shown in Figure S1. A closer inspection of the optical phonon modes at the Γ point reveals doubly degenerate branches along the Γ-A direction at approximately 2.44 THz and 3.89 THz. These modes involve coupled atomic displacements combining bond stretching and angular distortions, and reflect the intrinsic chiral symmetry of the trigonal structure. While such degeneracies are compatible with chiral lattice dynamics, we emphasize that a definitive identification of Weyl phonons^34^ would require an explicit evaluation of phonon Berry curvature and topological charges, which is beyond the scope of the present work. The observed phonon features therefore suggest, but do not by themselves establish, topologically nontrivial phononic behavior.

Much less explored are the 2D allotropes of tellurium predominantly found in the trigonal α-phase (α-Te), characterized by the $P\overset{-}{3}m1$ space group, and the monoclinic β-phase (β-Te), belonging to the *P*2/*m* space group with distinct zigzag and armchair directions. Tellurium propensity to form these 2D monolayers is attributed to its outer valence electron configuration and a potential Peierls instability, a distortion of the periodic lattice in a one-dimensional crystal that breaks its perfect translational symmetry, in this case, tellurium helical chains.^35,36^ This instability can drive a spontaneous structural transition toward energetically more favorable 2D configurations, resulting in the formation of α-phase, show in Figure 2a. This structure exhibits a lower total energy, than the β-phase shown in Figure 2b. Additionally, we investigate the following 2D-tellurium structures: Figure 2c (buckled pentagonal), Figure 2d (buckled kagome), Figure 2e (buckled square), Figure 2f (planar hexagonal), Figure 2g (Lieb-like), Figure 2h (planar kagome) and Figure 2i (planar square). These forms of tellurium are proposed here inspired by novel newly synthesized 2D square/rectangular^10^ and hexagonal^11^ phases, kagome metals,^37,38^ pentagonal bismuthene,^39^ graphene^40,41^ and phosphorene^42^ just to cite a few.

**Figure 2:**
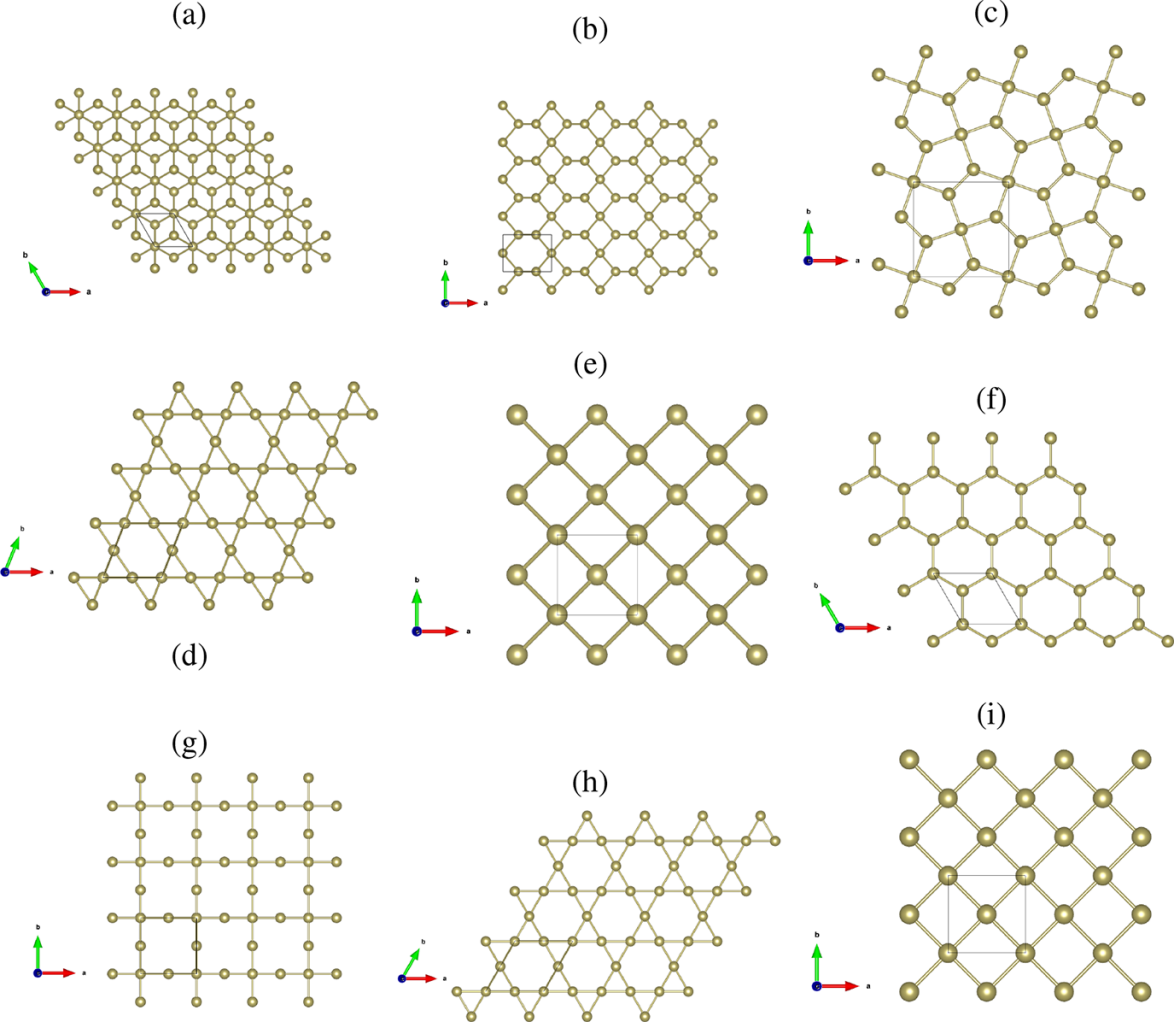
Relaxed crystal structures of 2D tellurium phases: (a) α-Te, (b) β-Te, (c) buckled pentagonal, (d) buckled kagome, (e) buckled square, (f) planar hexagonal, (g) Lieb-like, (h) planar kagome, and (i) planar square.

For α-Te, the calculated lattice parameters are *a* = *b* = 4.22 Å, with a Te–Te bond length of *d* = 3.03 Å, very similar to previous theoretical^6,43,44^ and experimental^36,45^ values. The β-tellurene phase exhibits lattice parameters *a* = 5.61 Å and *b* = 4.22 Å, featuring Te–Te bond lengths of *d* = 3.03 Å and *d* = 2.76 Å, similar to previous theoretical^6,44,46^ and experimental data.^47^ The buckled pentagonal structure has lattice parameters of 7.71 Å, the buckled kagome of 5.51 Å and the buckled square of 4.10 Å. This buckling leads to Te–Te bond lengths of 3.02, 2.96, and 3.03Å, respectively.

Topology is conditional on stabilization. The buckled kagome and buckled square tellurene lattices are found to host nontrivial *Z*_2_ = 1 topology arising from SOC-gapped near-crossings in the electronic structure. Phonon calculations indicate that these free-standing configurations exhibit soft modes, suggesting that their stabilization likely requires interaction with a substrate or external constraints. Importantly, similar kagome- and square-based tellurium phases have been experimentally realized on metallic substrates, supporting the physical relevance of these structures. Our results therefore establish the intrinsic topological character of these lattices, conditional on structural stabilization. Applying 5% strain modifies the band dispersions and shifts the band edges, but SOC continues to gap the near-crossings along Γ–X–M−Γ (Figure S6l). For the buckled structures, our results are comparable to previous ones for α-Te, β-Te,^48^ pentagonal,^49^ hexagonal.^11^ We emphasize the importance of the substrate: the DFT ground state is a square lattice, while in the experiment a rectangular reconstruction is found on Ni(111) substrate.^10^

Figure S2 shows the phonon dispersion curves of mechanically stable structures, with exception of the pentagonal structure that shows a couple of imaginary frequencies. The planar hexagonal, kagome, Lieb-like and square lattices in the absence of buckling are not mechanically stable (phonons not shown here), but we suggest nevertheless that these may be substrate-stabilized candidate phases.

In α-Te, one of the acoustic branches exhibits a soft mode (Figure S2a), indicating a possible dynamic instability under small perturbations. In β-Te, all three acoustic modes are softened, particularly along the armchair direction (Γ–X), and several optical branches show a noticeable reduction in energy (Figure S2b). This softening implies a higher susceptibility of these 2D structures to structural distortions or phase transitions when subjected to external perturbations. Although the buckled pentagonal, (Figure S2c), and buckled kagome phases show small imaginary frequencies, as seen in Figure S2d,e, it is be possible to stabilize these phases on an appropriate substrate. Strain induced by the substrate possibly introduce some electrostatic interaction and change the corrugation. This is consistent with experimental results for pentagonal,^49^ hexagonal,^11^ and rectangular lattices^10^ which are reported to be topological systems.

In β-Te, the isolated and nearly flat optical mode above 5 THz exhibits a noticeable hardening. This behavior can be attributed to modifications in interatomic bonding induced by reduced dimensionality. Additionally, several optical branches in the 1–2 THz range intersect with higher-energy acoustic phonons, suggesting enhanced phonon–phonon interactions and possible anharmonic effects in this phase. The modes in α-Te (a)–(e) presented in Figure S3 correspond to contraction and expansion motions within the *yz*-plane. In β-Te, the modes (f)–(i) shown in produce expansion and contraction within the *xy*-plane, reflecting in-plane lattice vibrations. The stiffer mode corresponds to an out-of-phase torsional motion of the atoms. Crossover points in the phonon dispersion are also observed at the high-symmetry K point.

We now turn our discussion to single-helix tellurium nanowires (Te–h). These one-dimensional structures can be obtained, for example, by decoupling the helical atomic chains that constitute bulk trigonal tellurium (Te–I),^50,51^ thereby preserving the intrinsic geometrical chirality and screw symmetry of Te–I at the single-chain level while removing the interchain packing present in the bulk crystal. The resulting quantum-confined geometry, combined with the strong spin–orbit coupling of tellurium, gives rise to distinctive one-dimensional electronic properties. We emphasize that the structural chirality of the helical chain should not be confused with chiral (sublattice) symmetry of the electronic Hamiltonian; in the present nanowires the latter is absent (see Figure S7 in Supporting Information and the details for Zak phase calculations.

Figure 3 shows the optimized structures of an ultrathin tellurium nanowire. The obtained lattice parameter is *c* = 5.67 Å with a Te–Te bond length of *d* = 2.74 Å in good agreement with previous theoretical results.^32,52^ From the phonon dispersion curves depicted in Figure S5, the absence of imaginary frequencies in Te–h along the high symmetry paths, indicating the possibility of local stability at low temperature, in agreement with previously reported theoretical data.^52^ Also, we see that the acoustic branches and one optical branch have softened, since their energies decrease compared to Te–I (Γ–A), suggesting potential structural phase transitions under small perturbations. On the other hand, the energy of some optical branches increased significantly (hardening). For example, the mode at 0.14 THz (Figure S5a) corresponds to torsional oscillations, similarly to the Te–I phase, but with a significantly lower eigenfrequency. This reduction is attributed to the absence of interchain interactions in the system. The Te–h phase hardens the modes that involve a combination of bond stretching and bending. The shorter Te–Te bond length in Te–h (2.74 Å) as opposed to bulk Te (2.90 Å) is consistent with this behavior.

**Figure 3:**
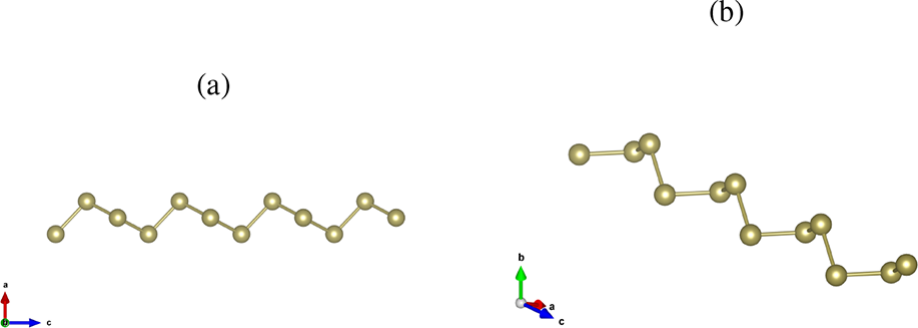
Helical geometry of the tellurium nanowire (Te–h). (a) Side view and (b) slightly rotated side view.

The cohesive energy serves as a key metric for evaluating the relative stability of various Te-based phases. Accordingly, the cohesive energy, *E*_coh_, is defined as $E_{\mathrm{coh}}=\frac{nE_{\mathrm{atom}}-E_{\mathrm{tot}}}{n}$ , where *E*_atom_ is the total energy of a single, isolated Te atom, *E*_tot_ is the total energy of the fully relaxed system, and *n* is the number of Te atoms in the structure.

According to Table 1, α-Te is identified as the most stable 2D phase, while the remaining phases are metastable with respect to α-Te. The smaller cohesive energy of α-Te compared to β-Te confirms its higher thermodynamic stability. Although β-Te is thermodynamically less stable than α-Te, the monoclinic structure may still be experimentally accessible under specific synthesis conditions.^9^ The buckled pentagonal, kagome, and square structures exhibit comparable cohesive energies, in agreement with experimental observations for these phases obtained under different substrates and growth conditions.^10,11,49^ This finding suggests that alternative tellurium phases can be stabilized depending on the experimental environment and synthesis parameters.

**Table 1:** Lattice Parameters *a*, *b,* and *c*, Interatomic Distances *d*_Te–Te_, Cohesive Energies *E*_coh_ and *C*_*v*_ for Tellurium Phases Calculated within GGA^a^

	lattice constant (Å)			*d*_Te–Te_ (Å)	*E*_coh_ (eV)	*C_v_* (J/K·mol)	*E*_g_ (eV)
phase	a	b	c				
Te–I	4.41	4.41	5.93	2.90	–2.75	73.68	0.30
α-Te	4.22	4.22		3.03	–2.61	73.50	0.75
β-Te	5.61	4.22		3.03, 2.76	–2.55	73.53	1.44
buckled pentagonal	7.71	7.71		3.02	–2.21	72.07	
buckled kagome	5.51	5.51		2.96	–2.30	72.70	
buckled square	4.10	4.10		3.03	–2.39	48.06	
Te–h			5.67	2.74	–2.38	73.07	2.23

a *E*_g_ calculated within HSE06.

For Te–h, the blue curve exhibits a steep slope, indicating a rapid increase in entropy with temperature (Figure S5b). This behavior arises from the enhanced vibrational degrees of freedom intrinsic to its one-dimensional structure. The results indicate that the primary contributions to the thermodynamic properties arise from in-plane atomic interactions as the dimensionality decreases from bulk to monolayer. Consequently, interlayer interactions—absent in 2D systems—appear to play only a minor role in determining the thermal stability of these structures.

The specific heat (*C*_*v*_) at *T* = 300 K is calculated to be around 73 J/(K·mol) for 3D, 2D and 1D phases (with exception of buckled square) shown in Figures S1, S4, S5 and Table S1. In comparison, graphene exhibits a much lower value of approximately 7 J/(K·mol).^53^ For phosphorene, the specific heat is not constant across its allotropes; for black phosphorene, it has been reported as 12.39 J/(K·mol) at room temperature.^54^ Similarly, for monolayer 2H-MoS_2_, the reported value is 61.12 J/(K·mol) at 300 K.^55^ These thermodynamic results, cohesive energy values, and dynamical stability analyses exhibit a consistent trend, reinforcing the conclusion that Te–I represents the most stable phase, followed by the 2D phases and the 1D Te–h nanowire.

The MLWF-HSE06 band structures calculated with and without spin–orbit coupling (SOC), shown in Figure S6j for Te–I, reveal an indirect electronic band gap located at the high-symmetry point H. The computed band gaps are 0.49 (0.30) eV with (without) SOC, in good agreement with the experimental value of 0.33 eV.^56^ These results classify Te–I as a narrow-gap semiconductor. Within the energy range from −1 to 0 eV, the valence band at the H point is 4-fold degenerate when SOC is neglected. The inclusion of SOC lifts this degeneracy, resulting in two nondegenerate states and one doubly degenerate state at lower energy. The region between the high-symmetry points L–H–A highlights features of the conduction band where doubly degenerate states exhibit linear dispersion—indicative of Weyl nodes located close to the Fermi level.

The states in this energy range primarily originate from lone-pair electrons derived from *p*_*x*_ orbitals. The six unoccupied states correspond to antibonding configurations dominated by *p*_*z*_–*p*_*y*_ orbital interactions. The projected band structure and partial density of states (PDOS), shown in Figure 4a,b, confirm that the *p*_*x*_ and *p*_*y*_ orbitals dominate near the Fermi level, while contributions from *p*_*z*_ orbitals remain negligible.

**Figure 4:**
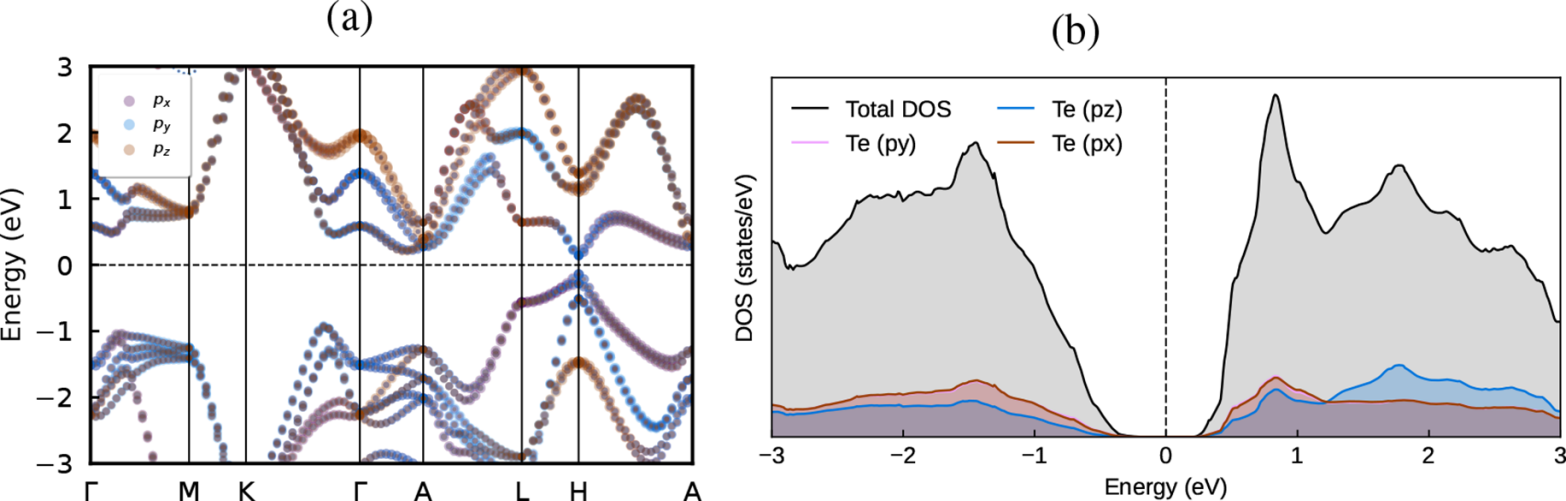
Orbital-projected (a) electronic band structure and (b) density of states of Te–I calculated within MLWF-HSE06+SOC.

Within the valence band, the PDOS reveals a substantial overlap between the *p*_*x*_ and *p*_*y*_ orbitals, indicating hybridization and strong orbital mixing. Moreover, the *p*_*z*_ orbital in this energy range exhibits a PDOS profile similar in shape to those of the *p*_*x*_ and *p*_*y*_ orbitals. A comparable trend is observed in the conduction bands within the 0–1 eV range, suggesting analogous orbital interactions at higher energies.

Given the identification of possible Weyl nodes in the band structure, we now turn to the analysis of spin textures. This investigation is crucial to confirm the topological nature of the material, since Weyl nodes are associated with characteristic spin–momentum locking, where the electron spin orientation is intrinsically coupled to its momentum direction.

Figure 5 shows indeed the two crossing points at the high-symmetry point H correspond to Weyl nodes. This identification is supported by the characteristic hedgehog-like spin texture in momentum space, where the spins align radially, creating spin patterns that act as “Berry monopoles” in momentum space and are connected with a defined chirality. A chirality charge of positive sign corresponds to a positive Chern number, while a Weyl node with a negative chirality charge has a negative Chern number. The magnitude of the spin components, as illustrated in the color bars in Figure 5, results directly from the effects of SOC. Consequently, in regions where SOC exerts a more significant influence on the electronic band structure, the spin components tend to display larger expectation values, indicating a stronger spin polarization.

**Figure 5:**
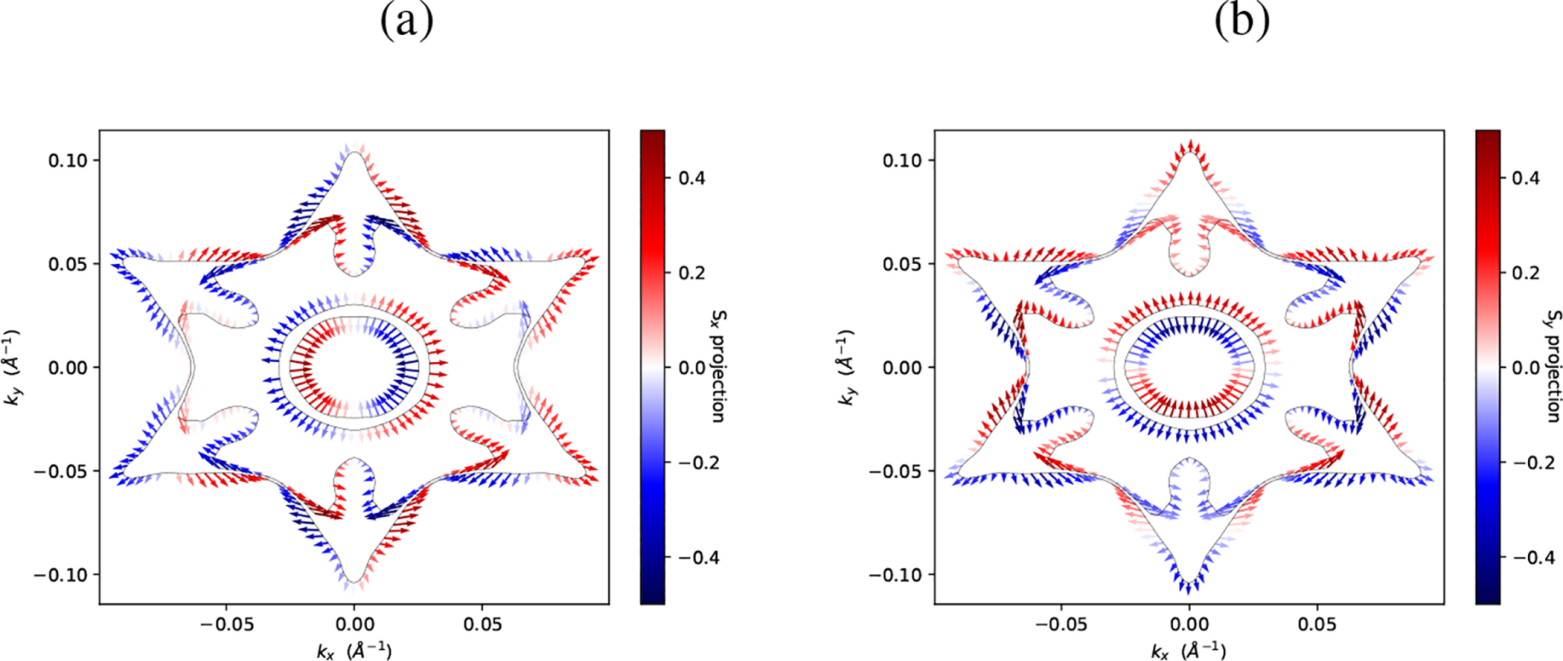
Spin textures of Te–I. (a) ⟨*S*_*x*_⟩ and (b) ⟨*S*_*y*_⟩ components of the trigonal phase at 0.9 eV. The color scale denotes the expectation values of the spin components.

Figure S6a,b present the electronic band structures of α-Te and β-Te, respectively, calculated using the MLWF-HSE06 exchange–correlation functional, both with and without spin–orbit coupling (SOC). The inclusion of SOC leads to a reduction in the band gap for both phases. Specifically, for α-Te, the band gap decreases from 1.04 to 0.75 eV upon inclusion of SOC, in excellent agreement with previous theoretical reports.^6,43,45^ Similarly, for β-Te, the band gap decreases from 1.77 to 1.44 eV with SOC, consistent with earlier theoretical studies.^6,45–48^

Upon inclusion of spin–orbit coupling (SOC), β-Te undergoes a transition from an indirect to a direct band gap at the Γ point, whereas α-Te retains its indirect gap. This band gap transition in β-Te has the potential to enhance the material optical absorption efficiency. SOC induces a significant reshaping of the valence bands in both monolayers, notably lifting the degeneracy at linearly dispersive crossing points. In the conduction band, β-Te exhibits a smaller degree of band splitting compared with α-Te. Furthermore, α-Te presents a quasi-flat band along the M−Γ–K direction, while β-Te displays a similar feature in the conduction band along the Γ–X–M path. The presence of such flat bands implies an accumulation of electronic states within a narrow energy range, typically leading to strong electronic correlations and enhanced carrier localization—properties of great interest for optical applications and potentially for superconductivity.

For β-Te, the *p*_*y*_ orbital provides the dominant contribution to the valence band maximum (VBM) near the Fermi level. In contrast, the conduction band minimum (CBM) exhibits a mixed contribution from both *p*_*y*_ and *p*_*x*_ orbitals, with the latter being slightly more prominent, as shown by the projected band structure in Figure 6. The total DOS indicates the presence of highly localized states in both monolayers: β-Te (Figure 6b) shows a slightly lower degree of delocalization compared to α-Te (Figure 6a). This behavior arises from quantum confinement when the dimensionality is reduced from three to two. The reduction in available electron degrees of freedom perpendicular to the monolayer forces the particles to occupy discrete energy levels within this confined region. The projected band structures of buckled pentagonal (Figure 6c), buckled kagome (Figure 6d), and buckled square (Figure 6e) lattices all exhibit metallic character. Among them, the buckled kagome lattice shows the highest DOS at the Fermi level, primarily originating from Te-*p* states.

**Figure 6:**
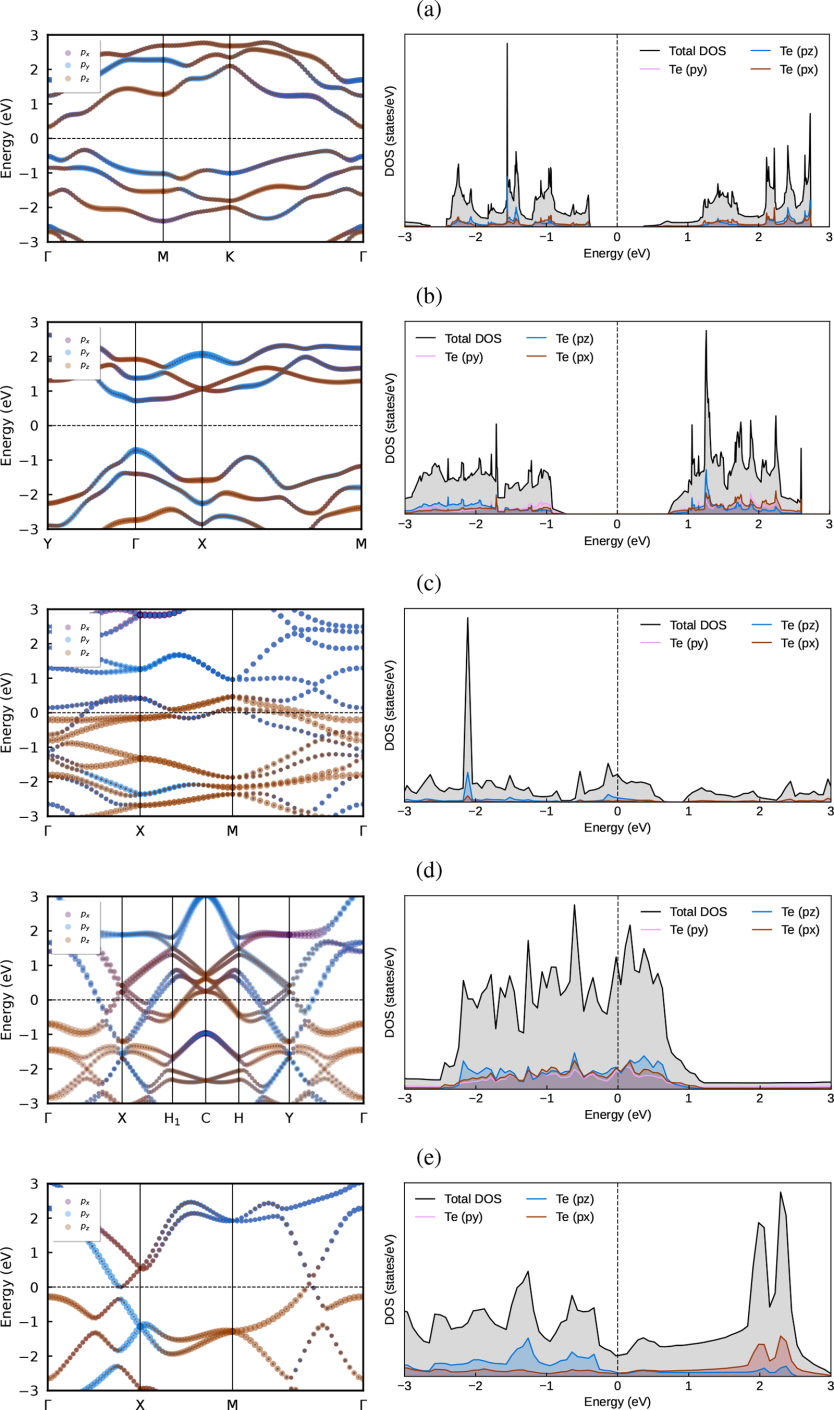
Orbital-projected electronic band structures and DOS for 2D tellurium phases calculated within MLWF-HSE06+SOC: (a) α-Te, (b) β-Te, (c) buckled pentagonal, (d) buckled kagome, and (e) buckled square. The contributions from *p*_*x*_, *p*_*y*_, and *p*_*z*_ orbitals are indicated by the thickness of the dots.

In order to confirm the presence or absence of nontrivial electronic states, a spin-texture analysis will be carried out to identify signatures of nontrivial spin behavior that may not be apparent from the band structures alone. Figure 7 reveals an intriguing behavior: despite the distinct spin patterns—tangential in the inner band and radial in the outer band for α-Te (Figure 7a), and predominantly radial for β-Te (Figure 7b)—no clear spin splitting is observed. This indicates that the bands remain degenerate even in the presence of SOC. Although spin degeneracy breaking is often associated with nontrivial electronic states, particularly in Weyl semimetals and topological insulators, it is important to note that topological properties may arise from mechanisms other than spin splitting. To conclusively determine the topological character, we compute both the Chern number and the $Z_{2}$ invariant. The results obtained using the MLWF–HSE06+SOC approach confirm that both α-Te and β-Te monolayers are topologically trivial.

**Figure 7:**
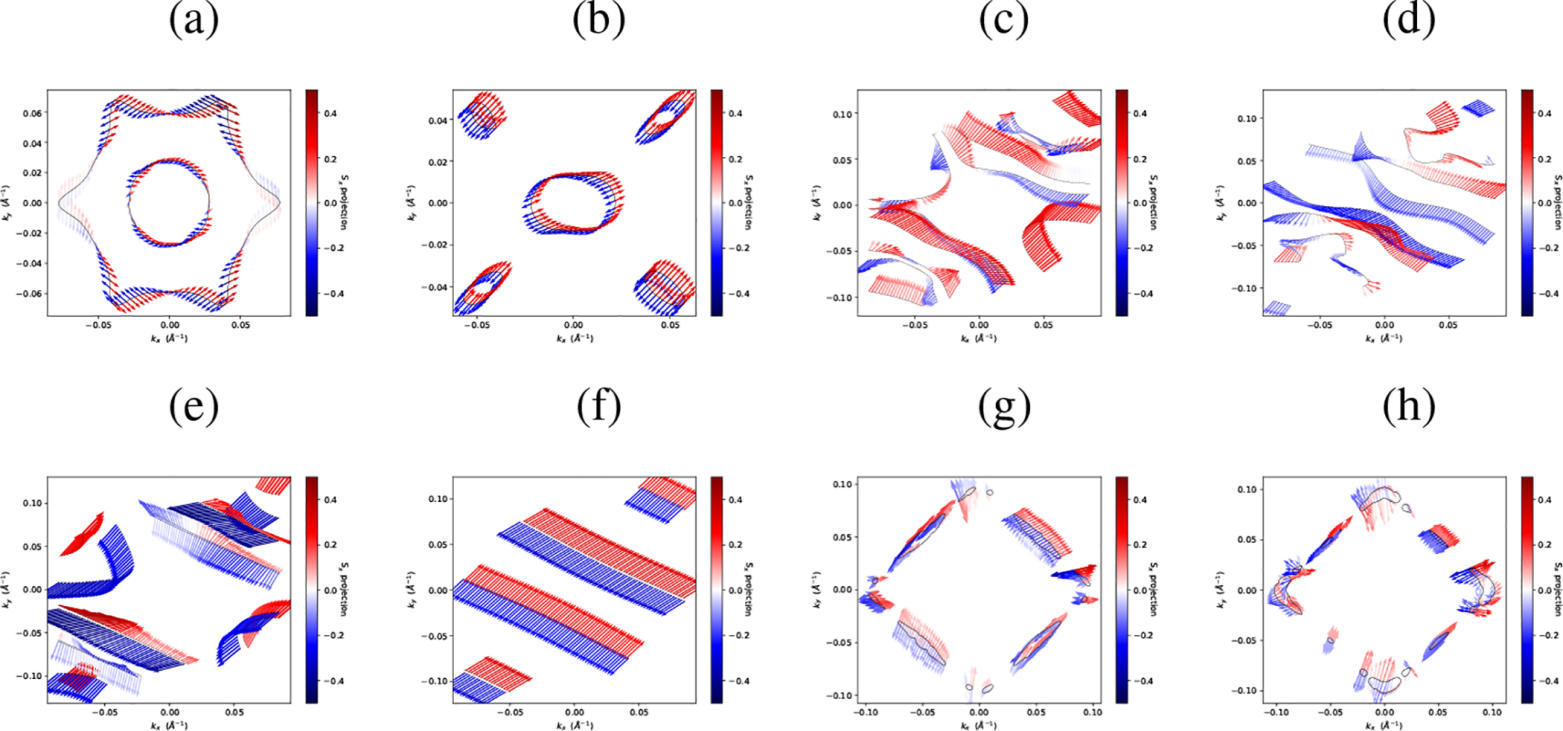
Spin textures of tellurium phases. ⟨*S*_*x*_⟩ component for (a) α-Te, (b) β-Te, (c, d) buckled pentagonal, (e, f) buckled kagome, (g, h) buckled square phase. The color scale denotes the expectation values of the spin components. All calculation performed within the MLWF-SE06+SOC.

This behavior is attributed to the fact that spatial inversion symmetry—α-Te belongs to the space group $P\overset{-}{3}m1$ , which contains an inversion center. On the other hand, β-Te belongs to the space group *P*2/*m*, which has both spatial and time-reversal symmetry inversion. This prevents the formation of Weyl nodes in the band structure. Likewise, the lack of SOC-induced band inversion precludes the characterization of these systems as topological insulators.

Nevertheless, it is important to note that nontrivial topological phases can arise under suitable external conditions. Topological phase transitions may be induced by perturbations such as mechanical strain, magnetic impurities, or doping. As discussed in ref 58, for instance, applying isotropic strain can drive tellurene from a trivial state into a topological phase. For completeness, the ⟨*S*_*x*_⟩ spin components of the: pentagonal phase are shown in Figure 7c,d, buckled kagome phase (Figure 7e,f), and buckled square phase (Figure 7g,h).

Figure 8a presents the band structure of the Te helicoidal nanowire (Te–h), computed using the MLWF-HSE06 functional both with and without SOC. The inclusion of SOC consistently narrows the band gap, reducing it from 2.49 to 2.23 eV. These values align well with previous theoretical reports.^32^ In both cases, the band structure displays predominantly flat dispersion. This combination of a finite band gap and quasi-flat bands makes Te–h an appealing material for photonic applications.^59^ Figure 8b shows the inclusion of SOC lifts band degeneracies in a manner similar to that observed in Te–I along the Γ–A direction. Along the high-symmetry path of Te–h, four band crossings are identified: two occurring at the same *K*-point—P1 in the valence band at −2.7, eV and P2 in the conduction band near 2, eV—and two additional crossings near the Γ-point, P3 at −2.7, eV and P4 around 2, eV. Figure 8c presents the PDOS for Te–h, showing that the *p*_*x*_, *p*_*y*_, and *p*_*z*_ orbitals contribute comparably to both the VBM and CBM. However, the CBM exhibits a more pronounced contribution from the *p*_*x*_ orbital. Due to quantum confinement, Te–h displays highly localized electronic states, which is reflected in the discrete features of the band structure and in the pronounced peaks observed in the DOS.

**Figure 8:**
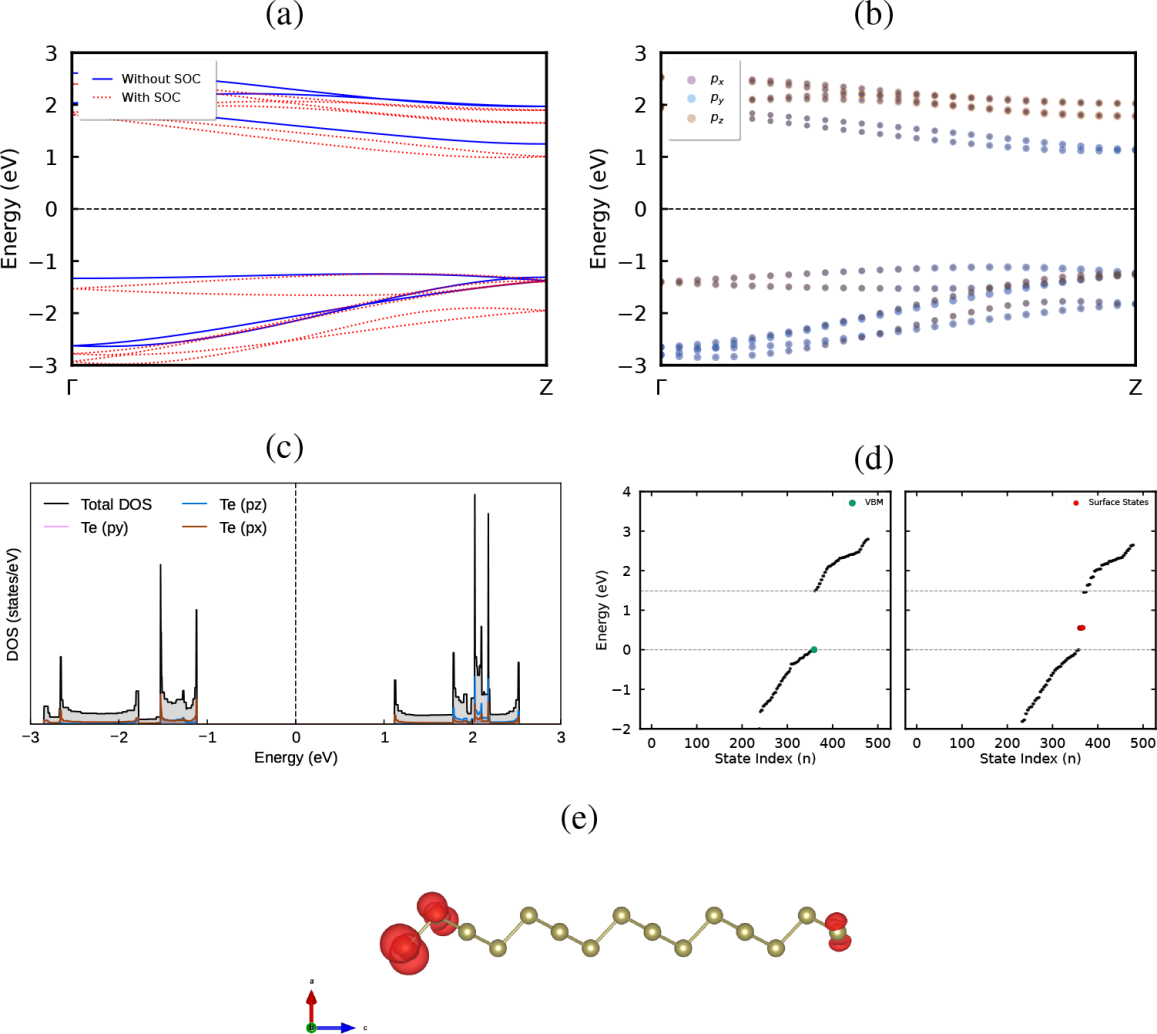
(a) Electronic band structure of the nanowire calculated without (gray) and with (colored) spin–orbit coupling (SOC), highlighting the pronounced SOC-induced modification of the confined spectrum. (b) Orbital-resolved representation of the SOC-included band structure, indicating that the states near the Fermi level are dominated by Te *p*-orbitals. (c) Total electronic density of states (DOS) of the nanowire, confirming the presence of a finite energy gap in the quantum-confined geometry. (e) Real-space charge-density isosurfaces of representative (d) in-gap states, revealing strong localization at the edge atoms of the nanowire.

Figure 8d illustrates the edge-derived states that lie within the nanowire band gap. The presence of highly localized states originating from the edge atoms is further highlighted in Figure 8e. The helical tellurium nanowire preserves the broken inversion symmetry and structural chirality of bulk trigonal Te, while introducing strong quantum confinement. In the presence of spin–orbit coupling, this reduced dimensionality leads to the emergence of in-gap electronic states that are strongly localized at the terminal atoms of the nanowire, as evidenced by the projected charge-density analysis. Although a strict $Z_{2}$ topological invariant is not formally defined for isolated one-dimensional systems, these edge-localized states can be naturally interpreted as boundary manifestations inherited from the higher-dimensional Weyl semimetal parent phase. The coexistence of time-reversal symmetry, strong SOC, and chirality stabilizes these boundary modes, distinguishing them from trivial dangling-bond states. The topological properties of the tellurium nanowire were analyzed within the modern theory of polarization. Since the system is one-dimensional and preserves time-reversal symmetry, no $Z_{2}$ topological invariant associated with quantum spin Hall phases can be defined. Instead, the relevant bulk quantity is the Berry (Zak) phase accumulated along the one-dimensional Brillouin zone. Because the nanowire lacks inversion and chiral symmetries, the Zak phase is not symmetry-quantized. Our calculations yield a Zak phase essentially equal to zero, corresponding to a vanishing bulk polarization (SI, Figure S7). This demonstrates that the periodic nanowire is topologically trivial in the normal (nonsuperconducting) state. Consistently, any end-localized states observed in finite nanowires originate from termination effects and are not protected by bulk topology.

The effective masses (Table S2) were extracted from the band structure near the inflection points at the VBM and CBM. Te–h effective masses are suggestive of potentially high mobility.^60–62^ Moreover, the modest reduction in electron and hole mobility in Te–h can help mitigate current leakage in nanoelectronic devices.^63^ In units of the free-electron mass, the electron effective mass of Te–h is 0.484, while the hole effective mass is 0.817 (see Table S2).

In Table 2, the topological invariant $Z_{2}$ for the 2D phases of tellurium is presented. The invariants are calculated following the method described in ref 26. The buckled kagome and buckled square phases exhibit nontrivial topology, whereas α-Te, β-Te, and the buckled pentagonal phase are topologically trivial. These topological classifications are consistent with the spin textures shown in Figure 7.

**Table 2:** $Z_{2}$ Topological Invariant Calculated within MLWF-HSE06

phase	$Z_{2}$	topological class	berry-curvature character
α	0	trivial	
β	0	trivial	
passivated hexagonal	1	nontrivial	valley-localized
buckled kagome	1	nontrivial	strong SOC-induced hotspots
buckled square	1	nontrivial	moderate, symmetry-localized
buckled square strained	1	nontrivial	valley-localized

Using experimentally reported lattice parameters,^11^ the planar hexagonal tellurene phase is found to exhibit semimetallic behavior and a nontrivial $Z_{2}=1$ index when SOC is included, consistent with previous experimental observations. However, our phonon calculations indicate that the free-standing planar hexagonal lattice is dynamically unstable. We therefore investigated stabilization mechanisms through strain engineering and surface functionalization. Under 5% isotropic in-plane strain, as well as under one-side hydrogen passivation, the system undergoes a transition to a gapped electronic state while preserving a nontrivial $Z_{2}=1$ invariant. In these stabilized configurations, the SOC-induced gap and the winding of the Wilson loop unambiguously identify a quantum spin Hall phase.

Similarly, one-side hydrogen passivation of the hexagonal phase yields a $Z_{2}=1$ phase (Figure S6k), depending on the Fermi-level position it can appear semimetallic, but the SOC-opened gap indicate QSH-type topology. These findings demonstrate that the topological features remain robust under both strain and surface functionalization. Therefore, controlling strain or chemical termination provides viable strategies to engineer and stabilize topological phases in two-dimensional tellurium.

We now turn our attention to the calculation of the tellurium effective masses, obtained from the slopes of the band structure near the VBM and CBM. The obtained values are shown in Table S2. Since the effective mass is inversely related to carrier mobility, the relatively low values found for Te–I suggest anisotropic electron and hole mobilities. For Te–I, the electron and hole effective masses are 0.614 and 0.335, respectively.

For the 2D phases, the effective masses for electrons (holes) in α-Te are 0.108 (0.135). In β-Te, the electron effective masses are 1.009 (along X) and 0.203 (along Y), while the hole effective masses are 0.368 (X) and 0.127 (Y), indicating strong transport anisotropy. Our results suggest that both α-Te and β-Te could have higher electron and hole mobilities than structurally or symmetrically similar materials such as 2H-MoS_2_ ^64,65^ and phosphorene.^66,67^ While α-Te exhibits higher mobilities for both charge carriers overall, β-Te shows pronounced anisotropy due to its geometry, with significantly reduced mobility along the armchair direction (Γ–X) compared to the zigzag direction (Γ–Y).

For the other phases, the buckled pentagonal structure yields effective masses of 0.220 (electron) and 0.172 (hole). The buckled square lattice is anisotropic, with electron masses of 0.100 (CBM−Γ) and 0.148 (CBM–X), and hole masses of 0.459 (VBM−Γ) and 0.239 (VBM–M). Finally, the hydrogen-passivated hexagonal phase is slightly asymmetric, with electron effective masses of 2.400 (Γ–M) and 2.310 (Γ–K), and a hole effective mass of 1.184 (VBM−Γ).

Figure 9 shows the spin texture of hydrogen passivated hexagonal tellurium at an energy *E*_F_ – 0.3 eV. The one-side H-passivated hexagonal tellurene exhibits clear in-plane spin-momentum locking and Rashba-type spin splitting. The constant-energy spin textures show nearly circular spin-split contours as well as pronounced hexagonal warping, indicating strong inversion asymmetry induced SOC and anisotropic spin polarization around Γ.

**Figure 9:**
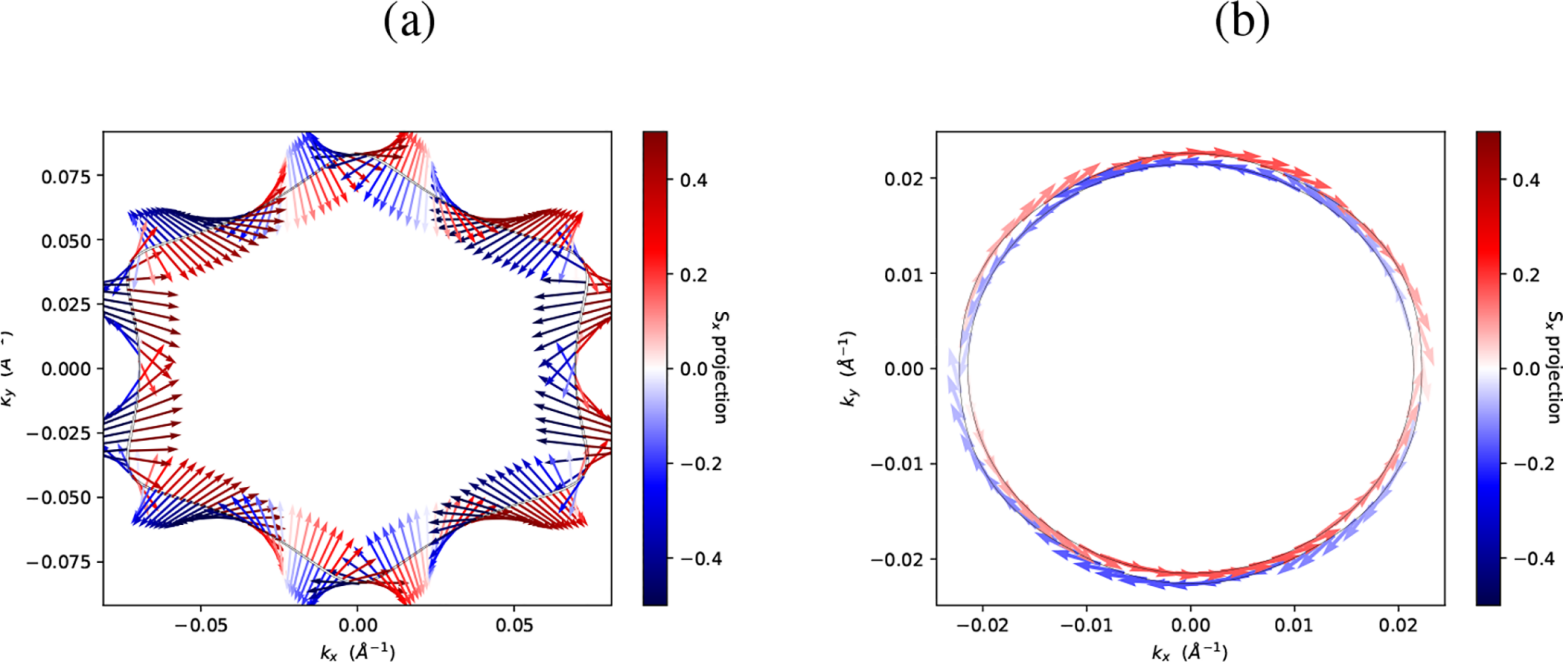
(a, b) Spin texture of hydrogen passivated hexagonal tellurium at *E*_F_ – 0.3 eV calculated within MLWF-HSE06+SOC.

The topological properties of the two-dimensional tellurene phases were characterized using the Chern number and the $Z_{2}$ invariant, both evaluated from the Berry curvature and the evolution of the occupied electronic subspace in momentum space. For systems that break time-reversal symmetry, the Chern number *C* is defined as the Brillouin-zone integral of the Berry curvature summed over all occupied bands,

$$C=\frac{1}{2\pi }\int_{\mathrm{BZ}}\Omega_{xy}\left(k\right)d^{2}k$$ 1

where Ω_*xy*_(**k**) is the Berry curvature summed over the occupied bands, Ω_*xy*_(**k**) = ∑_*n*∈occ_Ω_*n*,*xy*_(**k**). In our implementation, Ω_*n*,*xy*_(**k**) is evaluated using the Kubo (velocity-matrix) expression, as implemented in WannierTools,

Ωn,xy(k)=−2Im∑m≠n⟨unk|v^x|umk⟩⟨umk|v^y|unk⟩(εmk−εnk)2 2

where |*u*_*n* **k**_⟩ and ε_*n* **k**_ are the cell-periodic eigenstates and eigenvalues of the Wannier-interpolated tight-binding Hamiltonian *H*(**k**), and the velocity operators are obtained from *v̂*_α_ = (1/*ℏ*) ∂*H*(**k**)/∂*k*_α_. In time-reversal-symmetric systems, the Berry curvature satisfies Ω_*xy*_(**k**) = – Ω_*xy*_(−**k**), enforcing a vanishing total Chern number, although sizable local Berry-curvature contributions may still occur. For time-reversal-invariant two-dimensional systems, the relevant topological index is the $Z_{2}$ invariant, which distinguishes trivial insulators from quantum spin Hall phases. The $Z_{2}$ invariant was determined from the evolution of the Wilson loop, or equivalently the Wannier charge centers, of the occupied bands across the Brillouin zone. A nontrivial topological phase corresponds to an odd winding of the Wannier charge centers and yields $Z_{2}=1$ , whereas the absence of winding indicates a trivial phase with $Z_{2}=0$ .

The Berry curvature and Chern number were computed using tight-binding Hamiltonians constructed from MLWF ensuring an accurate interpolation of the electronic structure on dense **k**-point meshes. Spin–orbit coupling was included in all topological calculations, as it is essential for capturing the SOC-driven band inversions and avoided crossings responsible for nontrivial topology in tellurene.^25^

The topological behavior of tellurene polymorphs is shown to be highly sensitive to lattice geometry, buckling, and symmetry breaking, leading to a hierarchy of quantum phases within the same chemical composition. Among the phases examined here, α and β-tellurene are topologically trivial, whereas buckled kagome, buckled square, and one-side passivated hexagonal tellurene all realize nontrivial two-dimensional $Z_{2}=1$ topology. Results are shown in Table 2 and Figure 10.

**Figure 10:**
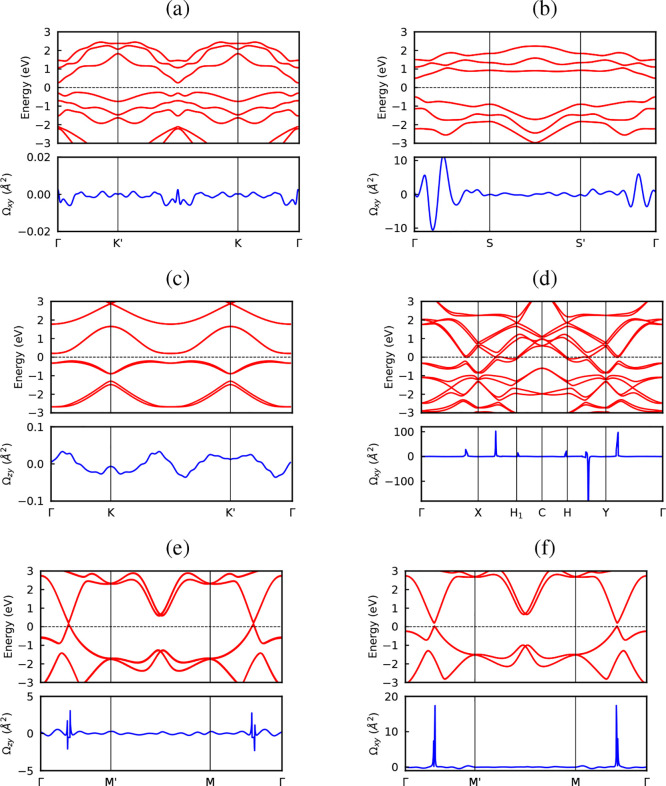
Berry curvature distributions calculated within the two-dimensional Brillouin zone for different tellurium phases: (a) α-tellurene, (b) β-tellurene, (c) one-sided hydrogen-passivated hexagonal tellurene, (d) buckled kagome tellurene, (e) buckled square tellurene, and (f) buckled square tellurene under 5% strain.

Along the Γ–K*′*–K−Γ path, the calculated Berry curvature Ω_*xy*_ of α-tellurene remains very small and close to zero over the entire energy window shown, with weak localized feature between K*′* and K. This behavior indicates the absence of pronounced Berry-curvature hot spots typically associated with band inversion or strong valley-contrasting responses. The near-cancellation of Ω_*xy*_ along the high-symmetry path is consistent with a topologically trivial, time-reversal-symmetric insulating phase ( $Z_{2}=0$ ) and implies a negligible intrinsic Hall-type contribution in the bulk. The small residual peak likely originates from a local avoided crossing or near-degeneracy in the band structure (or minor interpolation and *k*-mesh effects), rather than from a robust topological signature.

β-tellurene remains a trivial insulator ( $Z_{2}=0$ ) despite the presence of spin–orbit coupling. Although SOC induces band splittings and generates localized Berry-curvature features associated with avoided crossings, it does not alter the global band connectivity of the occupied manifold. No SOC-driven band inversion occurs, and the Wilson-loop evolution exhibits no topological winding. This demonstrates that Berry-curvature hotspots alone are insufficient to guarantee nontrivial $Z_{2}=1$ topology. Rather, a change in the global ordering of bands across the Brillouin zone is required.

In contrast, one-side passivated hexagonal tellurene provides a complementary route to QSH behavior through explicit breaking of out-of-plane symmetry. Passivation shifts the system toward a near-crossing regime in the absence of SOC, while SOC opens a bulk gap and stabilizes a $Z_{2}=1$ phase. Unlike the kagome and square lattices, the Berry curvature in this structure is predominantly valley-localized around the K and K′ points, reflecting the hexagonal symmetry and inversion asymmetry of the lattice. The resulting curvature pattern cancels globally under time-reversal symmetry but highlights the valley-resolved nature of the topological response, suggesting potential interplay between QSH and valley physics.

Buckled kagome tellurene exhibits nontrivial $Z_{2}$ topology of the occupied band. The kagome lattice naturally hosts a dense manifold of near-degenerate bands, and buckling enhances SOC-induced hybridization among them. In the absence of SOC, the system lies close to a multiband crossing regime, while SOC lifts these degeneracies and opens a bulk gap. The resulting Berry curvature is highly localized and unusually large in magnitude near SOC-gapped avoided crossings, reflecting the strong interband mixing inherent to the kagome geometry. Despite the extreme local curvature, time-reversal symmetry enforces a vanishing Chern number, and the nontrivial topology is instead encoded in the $Z_{2}=1$ invariant. This phase represents the most pronounced manifestation of SOC-driven topology among the structures considered.

Buckled square tellurene also realizes a $Z_{2}=1$ -nontrivial phase, but through a comparatively simpler mechanism. Here, SOC gaps symmetry-allowed near-crossings present in the non-SOC band structure, yielding a well-defined insulating manifold with nontrivial Wilson-loop winding. The Berry curvature is more moderate and spatially confined than in the kagome case, indicating fewer competing near-degeneracies. Importantly, the persistence of $Z_{2}=1$ under moderate strain demonstrates that the square-lattice nontrivial phase is not accidental but instead represents a stable topological regime tunable by lattice deformation.

These results establish that nontrivial $Z_{2}=1$ topology in tellurene is not an isolated phenomenon but emerges systematically when lattice geometry and symmetry place the system near a SOC crossing regime. The kagome lattice maximizes this effect through band multiplicity, the square lattice offers a simpler and strain-robust realization, and one-side passivation enables topology through symmetry breaking and valley selectivity. In contrast, β-tellurene lacks the necessary band reordering and remains topologically trivial. This comparative analysis highlights tellurene as a versatile platform for engineering two-dimensional quantum spin Hall phases through structural design rather than chemical substitution.

## 4 Conclusions

In this work, we conducted a comprehensive first-principles investigation of tellurium across its dimensional hierarchy—bulk Te–I, 2D monolayers, and 1D helical nanowires—addressing structural, electronic, vibrational, and topological properties with full inclusion of spin–orbit coupling (SOC).

We confirmed that bulk trigonal Te–I is dynamically and thermodynamically stable and hosts Weyl nodes arising from broken inversion symmetry and strong SOC. Two-dimensional α-Te and β-Te monolayers are shown to be stable semiconductors and topologically trivial within our calculations ( $Z_{2}=0$ ), with no SOC-driven band inversion in the occupied manifold; however, their strong SOC suggests they are promising candidates for topological phase transitions driven by external strain, doping, or magnetic perturbations.

Buckled kagome, buckled square, and one-side passivated hexagonal tellurene exhibit nontrivial two-dimensional $Z_{2}=1$ topology of the occupied bands, with the latter realizing a fully gapped quantum spin Hall phase and the former two representing incipient QSH regimes accessible upon tuning the chemical potential. The realization of a nontrivial $Z_{2}=1$ topology in metallic kagome and square tellurene highlights an experimentally realistic route to quantum spin Hall phases, where topology is established at the band-structure level and insulating behavior can be achieved through electrostatic or substrate-induced tuning. Moreover, the associated SOC-induced Berry-curvature hotspots suggest enhanced spin and charge transport responses even in the metallic regime. Together, these results establish tellurium as a uniquely tunable platform for topology engineering across dimensionality. By bridging 3D Weyl physics, 2D symmetry-modulated topological phases, and termination-induced edge-localized states in finite nanowires, our study provides a unified framework for designing tellurium-based topological matter. This work not only advances the fundamental understanding of symmetry-driven phases in chalcogen systems but also highlights the potential of Te nanostructures for next-generation topological electronics, spintronics, and optoelectronic devices.

## Supplementary Material



## Notes

Vibrational, thermodynamic properties and band structure of selected tellurium phases, Zak phase of one-dimensional tellurium nanowire, and effective masses of selected tellurium phases (PDF)
