# Supplementary material for: Interplay between Structural, Electronic, and Topological Properties in Low-Dimensional Tellurium
Source: ACS Omega. 2026 Mar 6;11(10):16355–68. doi: 10.1021/acsomega.5c12108 (PMC13000616; doi:10.1021/acsomega.5c12108)
Supplement: Supplementary file 1 [file ao5c12108_si_001.pdf]

Supplementary Information

Interplay between structural, electronic and topological properties in low-dimensional tellurium

Gabriel Elyas Gama Araújo* and Andreia Luisa da Rosa*

Federal University of Goiás, Institute of Physics, Campus Samambaia, 74960600 Goiânia, Brazil

E-mail: gabrielelyas@proton.me; andreialuisa@ufg.br

1 Thermal properties

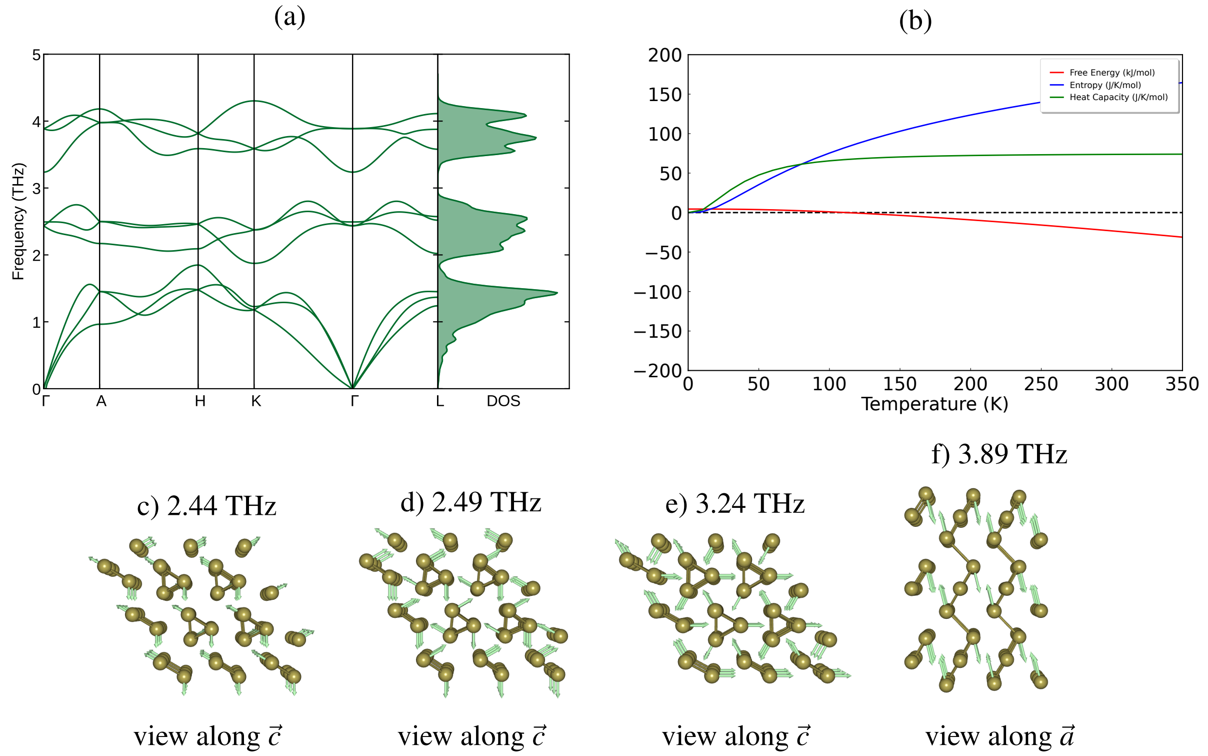

Figure S1: Vibrational and thermodynamic properties of trigonal tellurium (Te-I). a) Phonon dispersion curve and density of states and b) free energy, entropy and heat capacity. Selected phonon modes in Te-I: (c-e) projected along the $\vec{c}$-axis and (f) is viewed along the $\vec{a}$ axis.

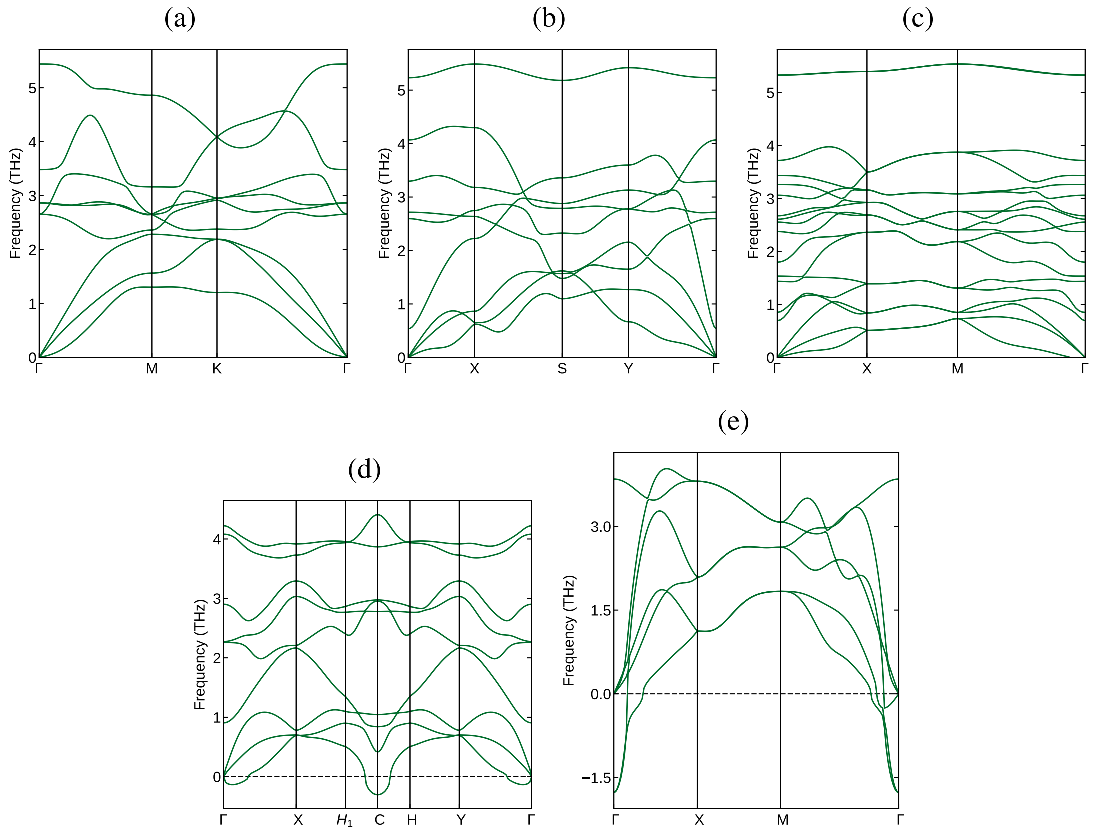

Figure S2: Phonon dispersion curves of 2D tellurene phases: a) α-Te, b) β-Te, c) buckled pentagonal, d) buckled kagome and e) buckled square.

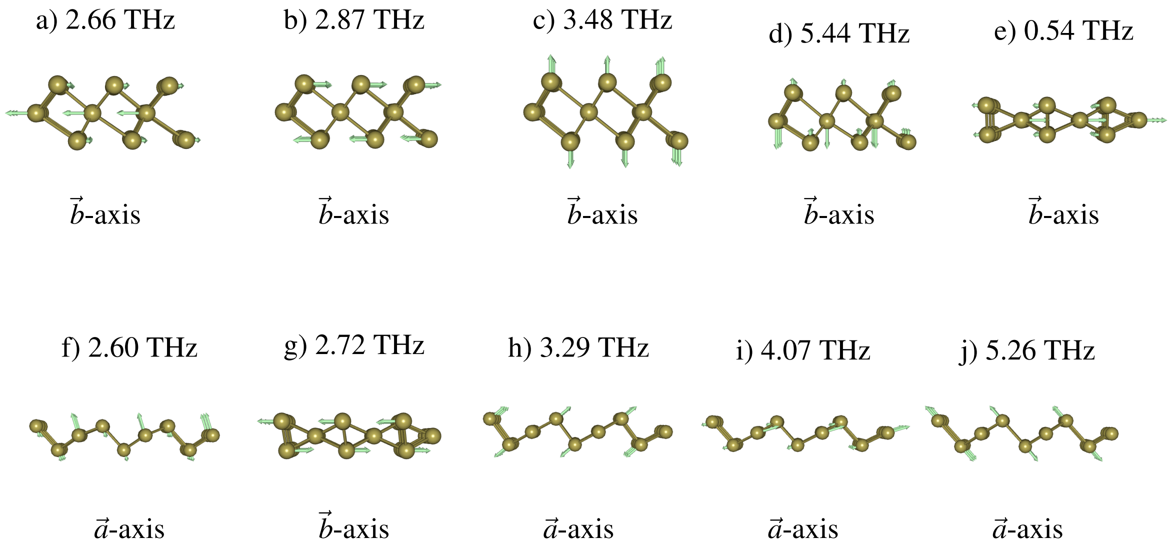

Figure S3: Selected phonon modes at Γ-point calculated withon GGA: (a-e) α-Te and (f-j) β-Te.

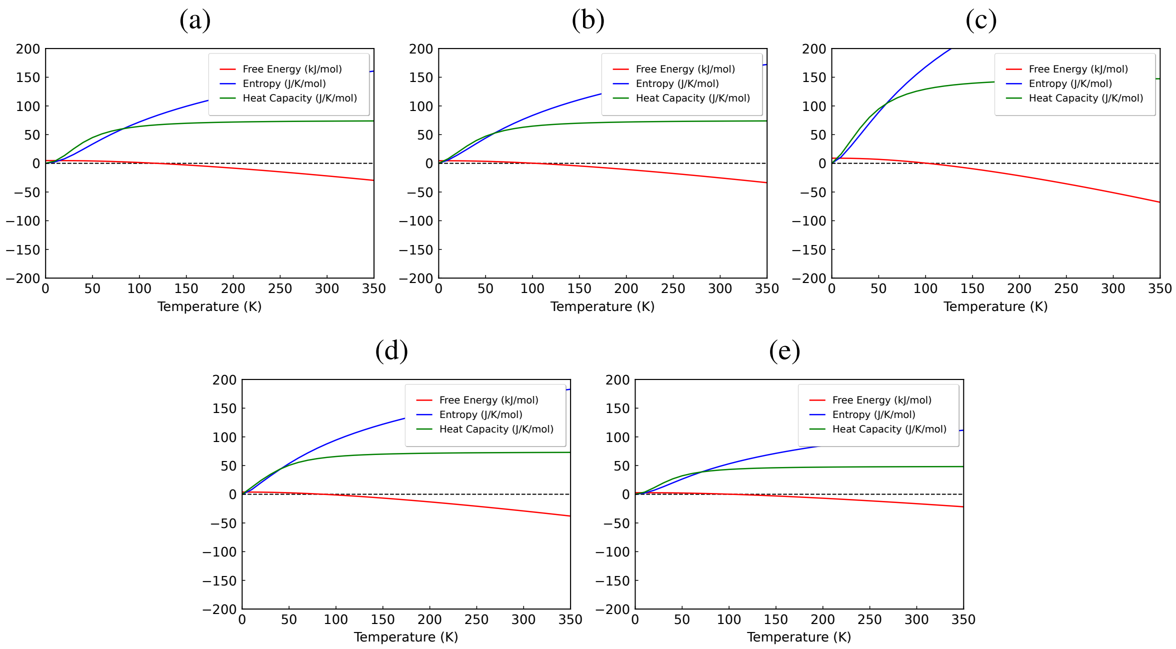

Figure S4: Thermal properties of tellurium 2D lattices: heat capacity (C_V), Helmholtz free energy (F), and entropy (S) calculated within GGA. a) α-Te, b) β-Te, c) buckled pentagonal, d) buckled kagome and e) buckled square.

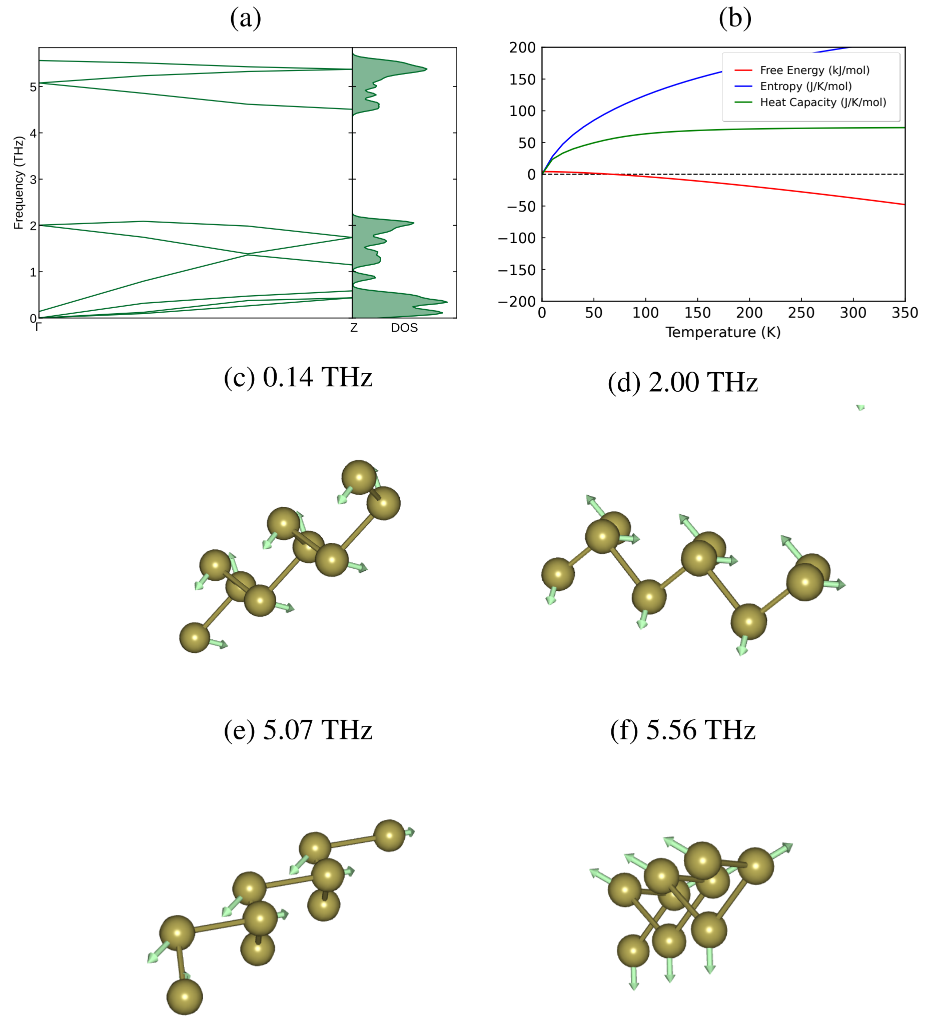

Figure S5: a) Phonon dispersion curve with phonon DOS and b) thermal properties of Te-h calculated within GGA. (c)-(f) Selected phonon modes at specific frequencies at Γ-point in Te-h.

Table S1: Entropy, free energy and specific heat at constant volume C_v at the dulong-Petit limit of tellurium phases.

phase	Free energy ($KJ/$mol)	Entropy ($J/K \cdot$mol)	C_v ($J/K \cdot$mol)
Te-I	-23.16	153.20	73.68
α-Te	-21.95	149.34	73.50
β-Te	-25.42	160.87	73.53
buckled pentagonal	-25.96	160.43	72.07
buckled kagome	-29.11	171.72	72.70
buckled square	-16.43	104.15	48.06
Te-h	-37.47	201.05	73.07

2 Role of SOC in the band structure

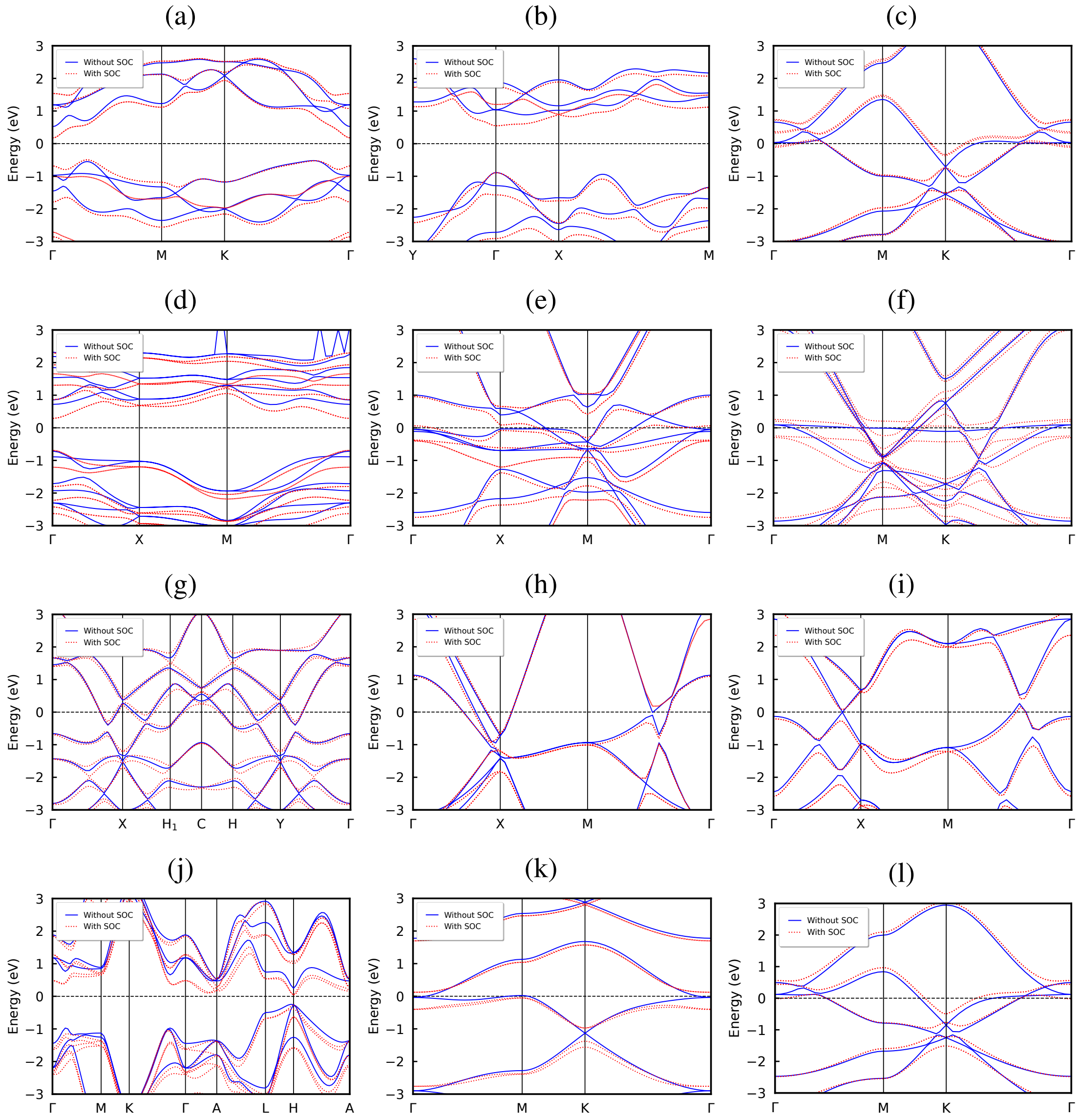

Figure S6: Band structure calculated within MLWF-TB/HSE06 of tellurium phases: a) α, b) β, c) hexagonal planar, d) Lieb-like, e) pentagonal buckled, f) planar kagome, g) buckled kagome, h) planar square, i) buckled square, j) trigonal Te-I, k) one-side hydrogen passivated hexagonal and l) hexagonal planar strained (5%). The highlighted region in k) reveals the emergence of Weyl nodes near the Fermi level, resulting from inversion symmetry breaking combined with strong SOC effects. Red (blue) lines are calculations with (without) spin-orbit coupling (SOC).

3 Topological characterization of one-dimensional systems

In one-dimensional crystalline systems, the topological classification of electronic bands differs fundamentally from that of two- and three-dimensional materials. For nonmagnetic systems with spin–orbit coupling that preserve time-reversal symmetry, the electronic structure belongs to

symmetry class AII in the Altland–Zirnbauer classification[1,2]. In contrast to higher dimensions, this symmetry class does not support a nontrivial $\mathbb{Z}_2$ topological insulating phase in one dimension. Consequently, a quantum spin Hall–type invariant cannot be defined for strictly one-dimensional, time-reversal-invariant band insulators. Instead, the relevant bulk quantity in one dimension is the Berry phase accumulated along the Brillouin zone, commonly referred to as the Zak phase[3]. This quantity is directly related to the electronic polarization and provides the appropriate framework for analyzing the topology of one-dimensional insulating systems.

For a one-dimensional periodic system, the Zak phase associated with the occupied electronic states is defined as

$$\gamma = i \sum_{n\in\text{occ}} \int_{\text{BZ}} \langle u_{nk} | \partial_k u_{nk} \rangle \, dk, \tag{1}$$

where $|u_{nk}\rangle$ are the cell-periodic Bloch functions and the integral is performed over the one-dimensional Brillouin zone.

Within the modern theory of polarization, the Zak phase is related to the bulk electronic polarization P via[4,5]

$$P = \frac{e}{2\pi}\gamma \quad (\text{mod } e), \tag{2}$$

where e is the elementary charge. Only the polarization modulo the electron charge is physically meaningful, reflecting the gauge freedom associated with the choice of unit cell.

The Zak phase is, in general, not a topological invariant, as its value depends on the choice of real-space origin and gauge. However, in the presence of spatial inversion symmetry or chiral symmetry, the Zak phase becomes quantized to values of 0 or π (mod 2π)[6,7]. In such cases, the quantized Zak phase serves as a symmetry-protected topological invariant and enforces the existence of robust boundary phenomena, such as topological end states.

Although Berry curvature plays a central role in the description of topological phases in higher-dimensional systems, it does not constitute a bulk topological invariant in strictly one-dimensional insulators. Nevertheless, Berry curvature distributions can provide useful qualitative information regarding band hybridization and spin–orbit–coupling effects. In particular, the absence of pronounced Berry-curvature peaks or singular features near the

band edges is consistent with the absence of band inversion and supports a topologically trivial characterization of the electronic structure. In one dimension, such Berry-curvature analyses should therefore be regarded as complementary diagnostics rather than as indicators of a topological phase.

The tellurium nanowire considered in this work is a one-dimensional, time-reversal-invariant system lacking inversion and chiral symmetries. Consequently, its Zak phase is not symmetry-quantized. The computed Zak phase is found to be essentially zero, indicating a vanishing bulk polarization. This establishes that the periodic nanowire is topologically trivial in the normal (non-superconducting) state. Accordingly, any localized states observed at the ends of finite nanowires are not protected by bulk topology and arise from termination-specific effects. The result for the Zak phase is shown in Fig.S7, calculated using VASP and WannierTools.

(a)

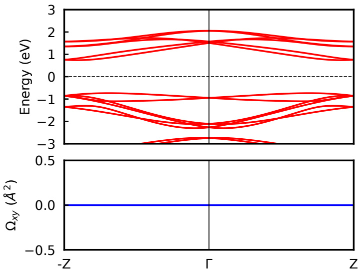

Figure S7: Zak phase of the one-dimensional tellurium nanowire. Berry phase accumulated by the occupied electronic states along the one-dimensional Brillouin zone. The Zak phase is found to be essentially zero, corresponding to a vanishing bulk polarization.

4 Effective masses

Table S2: Directional effective masses (m^*/m_e). For indirect band gap materials, paths are shown from the Valence Band Maximum (VBM) or Conduction Band Minimum (CBM). The fractional coordinates for these extrema are: Square buckled (VBM at [0.21, 0.21, 0.00]; CBM at [0.39, 0.00, 0.00]) and Hexagonal passivated (VBM at [0.49, 0.00, 0.00]; CBM at Γ).

	m* (m$_e$)	
Phases	Electron	Hole
α-Te	0.108	0.135
β-Te	1.009 (Γ →X)	0.368 (Γ →X)
	0.203 (Γ →Y)	0.127 (Γ →Y)
Pentagonal buckled	0.220	0.172
Square buckled	0.100 (CBM→ Γ)	0.459 (VBM→ Γ)
	0.148 (CBM→X)	0.239 (VBM→M)
Hexagonal passivated	2.400 (Γ →M)	1.184 (VBM→ Γ)
	2.310 (Γ →K)	

References

1 Schnyder, A. P. e. a. Classification of topological insulators and superconductors. *Phys. Rev. B* **2008**, *78*, 195125.

2 Ryu, S. e. a. Topological insulators and superconductors: Tenfold way. *New J. Phys.* **2010**, *12*, 065010.

3 Zak, J. Berry's phase for energy bands in solids. *Phys. Rev. Lett.* **1989**, *62*, 2747–2750.

4 King-Smith, R. D.; Vanderbilt, D. Theory of polarization of crystalline solids. *Phys. Rev. B* **1993**, *47*, 1651–1654.

5 Resta, R. Macroscopic polarization in crystalline dielectrics. *Rev. Mod. Phys.* **1994**, *66*, 899–915.

6 Resta, R. Manifestations of Berry's phase in molecules and condensed matter. *J. Phys.: Condens. Matter* **2000**, *12*, R107–R143.

7 Hughes, T. L.; Prodan, E.; Bernevig, B. A. Inversion-symmetric topological insulators. *Phys. Rev. B* **2011**, *83*, 245132.
